# Neutrophil extracellular traps and long COVID

**DOI:** 10.3389/fimmu.2023.1254310

**Published:** 2023-09-27

**Authors:** Areez Shafqat, Mohamed H. Omer, Ibrahem Albalkhi, Ghazi Alabdul Razzak, Humzah Abdulkader, Saleha Abdul Rab, Belal Nedal Sabbah, Khaled Alkattan, Ahmed Yaqinuddin

**Affiliations:** ^1^ College of Medicine, Alfaisal University, Riyadh, Saudi Arabia; ^2^ School of Medicine, Cardiff University, Cardiff, United Kingdom

**Keywords:** neutrophils, neutrophil extracellular traps, COVID-19, long covid, thrombosis, autoimmunity, fibrosis, inflammation

## Abstract

Post-acute COVID-19 sequelae, commonly known as long COVID, encompasses a range of systemic symptoms experienced by a significant number of COVID-19 survivors. The underlying pathophysiology of long COVID has become a topic of intense research discussion. While chronic inflammation in long COVID has received considerable attention, the role of neutrophils, which are the most abundant of all immune cells and primary responders to inflammation, has been unfortunately overlooked, perhaps due to their short lifespan. In this review, we discuss the emerging role of neutrophil extracellular traps (NETs) in the persistent inflammatory response observed in long COVID patients. We present early evidence linking the persistence of NETs to pulmonary fibrosis, cardiovascular abnormalities, and neurological dysfunction in long COVID. Several uncertainties require investigation in future studies. These include the mechanisms by which SARS-CoV-2 brings about sustained neutrophil activation phenotypes after infection resolution; whether the heterogeneity of neutrophils seen in acute SARS-CoV-2 infection persists into the chronic phase; whether the presence of autoantibodies in long COVID can induce NETs and protect them from degradation; whether NETs exert differential, organ-specific effects; specifically which NET components contribute to organ-specific pathologies, such as pulmonary fibrosis; and whether senescent cells can drive NET formation through their pro-inflammatory secretome in long COVID. Answering these questions may pave the way for the development of clinically applicable strategies targeting NETs, providing relief for this emerging health crisis.

## 1 Introduction

Long COVID (LC), also called post-acute sequelae of SARS-CoV-2 infection, encompasses the persistent symptoms following COVID-19. The Centre for Disease Control and Prevention (CDC) defines LC as symptoms lasting beyond four weeks post-SARS-CoV-2 infection, while the World Health Organization (WHO) describes it as the continuation or development of new symptoms three months following the initial infection, lasting for at least 2 months without any alternative explanation. LC is a multisystem disorder encompassing a multitude of clinical presentations including respiratory and cardiac dysfunction, neuropsychiatric disturbances, and hypercoagulability (1–3).

The prevalence of LC is rising, with an estimated 65 million people affected. This comprises 10-30% of non-hospitalized cases, 50-70% of hospitalized cases, and 10-12% of vaccinated-infected individuals (1–3). Hence, COVID-19 patients of all severities appear to be affected by LC. However, these figures likely underestimate the true prevalence and incidence of LC due to underreporting and undocumented cases. A substantial proportion of LC patients report significant impairments in their quality of life with an inability to perform tasks of daily living (4). Unfortunately, no effective treatment exists for LC, with a need for further high-quality and methodologically robust clinical trials (5). Viable therapeutic targets for long COVID are also lacking, partly due to the multitude of pathophysiological mechanisms that have been identified and a poor understanding of which of these mechanisms is causally linked to LC versus those that simply modulate its biology. Nevertheless, theories of LC pathophysiology converge on a chronic inflammatory response involving both the innate and adaptive arms of host defense (6, 7).

Neutrophils, the most abundant circulating leukocytes, constitute an essential component of innate immunity and are the first responders to sites of acute inflammation. They contribute to host defense by phagocytosis, pro-inflammatory cytokine production, degranulation, and the formation of neutrophil extracellular traps (NETs) (8, 9). Exaggerated neutrophil responses, particularly the production of NETs, have been implicated in the pathogenesis of acute COVID-19. NETs are web-like structures composed of cytosolic and granular neutrophil proteins embedded in a meshwork of either nuclear or mitochondrial neutrophil DNA (10–12). Various strategies targeting NETs have emerged as potential approaches to alleviate their pathological effects in COVID-19 and other autoimmune and inflammatory disorders (13–16).

Studies have indicated that the persistent release or impaired clearance of NETs after COVID-19 resolution may be linked to the pathogenesis of LC. This review explores the potentially disease-mediating role of NETs in the multi-faceted pathophysiology of LC.

## 2 Neutrophil extracellular traps

### 2.1 Production

**Figure 1:**
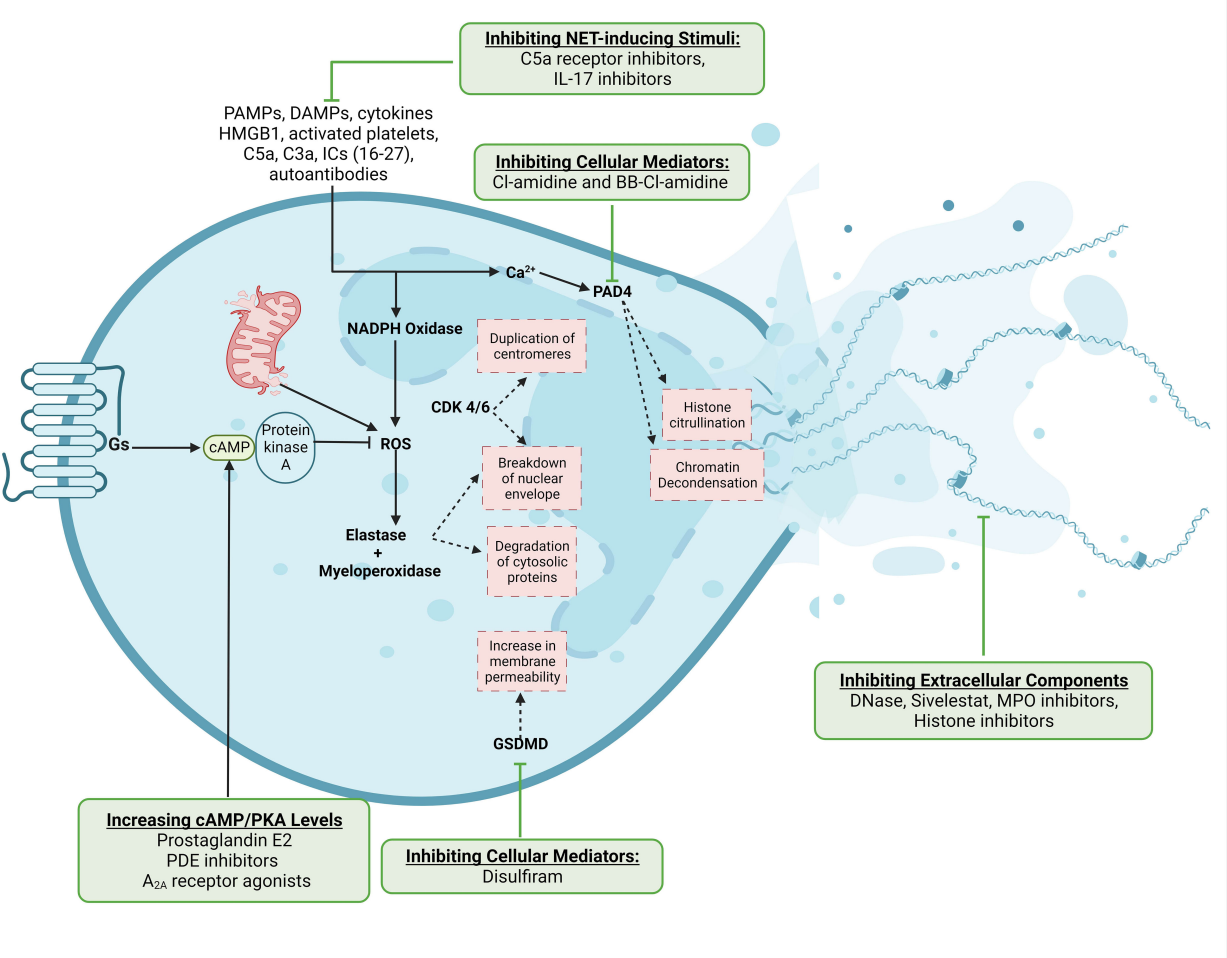
Mechanisms of NET production and potential therapeutic targets. NET-inducing stimuli converge on the production of reactive oxygen species, which activate neutrophil proteases and enzymes to cause downstream nuclear envelope breakdown and chromatin decondensation. The release of NETs either involves cell death with the extrusion of DNA-bound proteins or the exocytosis of NET components through the cell membrane from viable neutrophils. We have also indicated steps in this pathway which have been therapeutically targeted. This Figure was created using BioRender.com.

NET production, traditionally referred to as NETosis, was initially believed to occur exclusively through the pyroptotic cell death of neutrophils, resulting in the extrusion of nucleic acids and proteins into the extracellular space (**Figure 1**). Through a process called vital NET formation, however, neutrophils can release their contents and remain viable thereafter.

Various stimuli have been reported to induce NET formation, including microbial pathogen-associated molecular patterns (PAMPs) and their virulence factors, pro-inflammatory cytokines, damage-associated molecular patterns (DAMPs) like high-mobility group box-1 (HMGB1) and ATP, activated platelets, complement proteins C5a and C3a, autoantibodies, and immune complexes (17–28). These stimuli converge on increasing cytosolic calcium concentrations, leading to the activation of NADPH oxidase and the generation of reactive oxygen species (ROS) like superoxide and hydrogen peroxide—a process termed the respiratory burst (29). ROS molecules activate neutrophil proteases such as neutrophil elastase (NE) and myeloperoxidase (MPO) (30). Mitochondrial ROS can trigger similar processes but in a NAPDH oxidase-independent manner (31). NE and MPO then degrade the nuclear envelope, cleave numerous cytosolic proteins, and de-condense nuclear chromatin (30). The cell cycle proteins cyclin-dependent kinases 4 and 6 (CDK4/6) can also be repurposed to aid in nuclear envelope breakdown (32). Proteolytic cleavage of the pore-forming protein gasmerdin D (GSDMD) by caspases, NE, and MPO results in its activation and subsequent translocation to the cell membrane, increasing its permeability and augmenting the intracellular calcium flux (33, 34). Concomitantly, the activation of peptidylarginine deaminase-4 (PAD4), a calcium-dependent enzyme, catalyzes histone citrullination, disrupting the electrostatic attractions between DNA and histones and leading to chromatin decondensation (35). Ultimately, chromatin disassembly and compromised cell surface and nuclear membrane integrity lead to the release of DNA and proteins as NETs.

There is no singular marker for NETs, but extracellular DNA, NE, MPO, and citrullinated histone (cit-H3) are frequently used as surrogate markers. It is worth noting that numerous controversies have arisen regarding the exact relevance of several of the above-mentioned mediators. For instance, NET production in response to inflammasome signaling was previously thought to involve GSDMD-dependent processes (36), but recent GSDMD knockout experiments suggest a dispensable role of GSDMD in NET production (37). Similar results have been observed in NADPH and PAD4 knockout experiments (38, 39). These findings indicate that redundant mechanisms of NET production exist and call into question the exact functional relevance of many of these proteins and, by extension, their therapeutic value. Idiosyncratic, stimulus-dependent mechanisms likely exist, the elucidation of which may enable context-specific therapeutic manipulations. These controversies and others are covered in more detail elsewhere (see (40)).

From a metabolic standpoint, neutrophils have relatively few mitochondria compared to lymphocytes and macrophages and so rely predominantly on ATP acquired through glycolysis (41). The extrusion of NETs appears to also be majorly dependent on glycolysis, such that stimulating NET production *in vitro* by PMA under low glucose conditions causes neutrophils to lose their characteristic polymorphic nuclear architecture but does not result in the extrusion of NETs (42). Similarly, conditional deletion of the mitochondrial protein optic atrophy-1 (OPA1) in mice inhibits mitochondrial complex I activity, which decreases NAD^+^ availability for the glycolytic pathways and hence inhibits NETosis, with these models consequently displaying a subdued antibacterial response to *Pseudomonas aeruginosa* infections (43). It was later described that the activity of lactate dehydrogenase (LDH) and lactate production are essential for NET release, and the mechanism by which PMA induces NETosis involves LDH induction (44). Accordingly, sodium oxamate, an LDH inhibitor, attenuates NET production in mouse models of sepsis (44). Extracellular acidosis was recently shown to inhibit glycolysis and lactate production, thereby attenuating NETosis (45). The metabolic regulation of NET production seems, therefore, to be strictly dependent on glycolysis and lactate production and can be perturbed in systemic conditions where extracellular nutrient and metabolite levels are altered. However, whether therapeutically manipulating neutrophil metabolism can alter NETosis and allow us to modulate the pathogenesis of various disease states is not understood.

### 2.2 Therapeutic targets

Several mediators involved in NET production constitute therapeutic targets (**Figure 1**). For simplicity, we can divide NET-targeting strategies into four groups (1): inhibiting NET-inducing stimuli, such as pro-inflammatory cytokines, platelets, DAMPs like HMGB1, and complement proteins (2); inhibiting cellular mediators of NETosis, including ROS, PAD4, and GSDMD (3); the extracellular effectors of NETs, including NE, MPO, and histone inhibitors; and (4) augmenting the degradation of NETs through DNase-1. There are drugs in clinical use that inhibit NET-inducing stimuli, such as C5a receptor antibodies for ANCA-associated vasculitides and IL-17 inhibitors for psoriasis (46, 47). Other than these drugs, however, translational research on many of the other targets is lacking, and the ones that have been tested often lack a substantial clinical benefit. For example, the NE inhibitor sivelstat failed to significantly improve the outcomes of patients in clinical trials (48). The PAD4 inhibitors Cl-amidine and BB-Cl-amidine have shown promise in several animal models but lack human data (49–51). Dnase-1, which is FDA-approved for use as an inhalant in cystic fibrosis (CF) patients (52), is the only drug specifically targeting NETs approved for use. Recent studies have demonstrated the benefit of DNase-1 in improving outcomes of hospitalized patients with COVID-19-related ARDS, although validation in larger randomized studies is awaited (53–55). Disulfiram, commonly used for alcohol use disorder, can inhibit GSDMD pore formation and reduce NET burden and fibrosis in hamsters with COVID-19 (56, 57), but it has not been clinically studied for this indication. The cyclic adenosine monophosphate (cAMP)/protein kinase A (PKA) signaling pathway also suppresses NET production by inhibiting the oxidative burst. Therapeutically, this pathway can be leveraged by prostaglandin E_2_, cAMP analogs, phosphodiesterase inhibitors, and adenosine A_2A_ receptor agonists to increase intracellular cAMP levels and suppress NETosis (58–60). Overall, the COVID-19 pandemic has highlighted the abundance of potential targets for NET inhibition, but a glaring paucity of applicable medications whose clinical benefit has been substantiated.

## 3 Neutrophils in acute COVID-19

### 3.1 SARS-CoV-2 infection

SARS-CoV-2 is an enveloped positive-sense single-stranded RNA virus with a helical nucleocapsid belonging to the coronavirus family. Its viral genome encodes four structural proteins, including a surface spike (S) protein, which facilitates viral cell entry and membrane fusion (61). The S protein is composed of two subunits, S1 and S2. The S1 subunit contains the receptor-binding domain (RBD) which binds to angiotensin-converting enzyme 2 (ACE2) on host cell surfaces initially of the nasopharyngeal epithelium (62, 63). This initial interaction is established in the upper respiratory tract and is followed by subsequent seeding to the lower respiratory tract (64).

After binding to ACE2, the S protein undergoes cleavage by the transmembrane protease serine 2 (TMPRSS2) (62). Cleavage exposes the fusion peptide of the S2 subunit, resulting in the fusion of viral and host cell membranes (65). Following successful cellular infection, essential viral proteins are synthesized, and progeny SARS-CoV-2 virions are released via exocytosis to infect other cells. Type II pneumocytes are the primary ACE2-expressing cells in the alveolar epithelium, hence serving as the primary target of SARS-CoV-2 infection in the lower respiratory tract (66, 67).

Tissue-resident alveolar macrophages represent the first line of innate immune defense against SARS-CoV-2, recognizing SARS-CoV-2 PAMPs through various pattern-recognition receptors (PRRs), and triggering the production of pro-inflammatory cytokines and chemokines (68–70). This initial innate response is crucial in determining the severity of COVID-19 (71, 72). The timely production of type I and III interferons and cytotoxic T-cell responses is associated with mild-to-moderate infection (73–75). In contrast, severe disease arises from an impaired interferon and T-cell response coupled with an exaggerated innate neutrophil response (73–75). This dichotomy in the immune response plays a pivotal role in determining the severity of COVID-19 (71, 72). Clinically, the majority (~80%) of individuals infected with COVID-19 experience either asymptomatic or mild disease, with around 15% necessitating oxygen support, while 5% develop septic shock, ARDS, and multi-organ failure requiring aggressive treatments in intensive care units (ICUs) (76).

### 3.2 Neutrophil responses in COVID-19

Any discussion on the role of NETs in a specific disease is incomplete without describing the contextualized perturbations observed in the cells responsible for their production. This section delves into the current knowledge surrounding numerical and functional changes in neutrophils that ensue after SARS-CoV-2 infection. We focus on three key aspects: neutrophil alterations in the circulation, bronchoalveolar lavage (BAL), and histopathologic examination of lung autopsies from deceased COVID-19 patients.

Neutrophilia with an elevated neutrophil-to-lymphocyte ratio is a salient feature of severe COVID-19 (77). The degree of neutrophilia correlates positively with disease severity, being the highest in severe cases. Nonetheless, neutrophilia in COVID-19 is orders of magnitude less pronounced than that seen in bacterial ARDS (78). Notably, circulating neutrophils isolated from individuals with severe COVID-19 exhibit pronounced activated phenotypes (79), indicating that the disease not only induces numerical alterations but also significantly impacts the phenotype of the neutrophil compartment.

Recent research utilizing single-cell analysis has shed light on the transcriptional diversity of neutrophil populations (80, 81). A subset that has attracted considerable attention is interferon (IFN)^active^ neutrophils, which are thought to be primed for infection control (82). These subsets also appear in COVID-19, but their exact significance is still unclear (discussed below). Another way of classifying neutrophils is based on their density into low-density neutrophils (LDNs) and normal-density neutrophils (NDNs). LDNs are functionally heterogeneous groups of neutrophils but are generally considered more immature cells that produce higher levels of pro-inflammatory cytokines and NETs (83), with their emergence in autoimmune disorders like systemic lupus erythematosus (SLE) being associated with disease pathogenesis and severe clinical phenotypes (84, 85).

In the circulation, analyses of whole blood and PBMCs have demonstrated the emergence of immature neutrophil populations in severe—but not mild—COVID-19 (86, 87). This finding indicates the activation of emergency myelopoiesis, although the presence of immature neutrophils is not as significant as in cases of bacterial ARDS (78). Both immature and mature neutrophils display transcriptional signatures consistent with an activated state, characterized by upregulated calprotectin (i.e., S100A8/A9) expression and enhanced NET production (78, 87–89). Severe COVID-19 is also associated with the expansion of immunosuppressive clusters of neutrophils expressing the immune checkpoint molecule PD-L1 (87, 90–92), which declines in individuals recovering from severe disease. Another immunomodulatory cluster of neutrophils associated with severe disease are IFN^active^ neutrophils (87, 92), which are depleted by dexamethasone treatment, suggesting their involvement in the immunopathogenesis of severe COVID-19 (92). However, IFN^active^ neutrophils also occur in mild COVID-19, which is part of a coordinated interferon-stimulated gene (ISG) response across all major immune cell populations in the blood, whereas these ISG-expressing cells are systematically absent in patients with severe disease (93), consistent with the globally blunted type I and III IFN response characteristic of severe COVID-19 (73–75). These findings indicate that the presence of IFN^active^ neutrophils is not a necessary component of severe disease. The topic of neutrophil heterogeneity in COVID-19 has been detailed by other reviews (94, 95). Nonetheless, many of the current studies on this topic infer distinct functionality from the transcriptomic data rather than directly observing whether distinct subsets differentially impact COVID-19 pathogenesis. Subset-specific manipulations are required to further current findings.

Findings regarding neutrophil numbers and phenotypes in bronchoalveolar lavage (BAL) are largely concordant with those observed in the circulation. Numerically, mild COVID-19 does not exhibit a significant neutrophilic infiltrate, which progressively increases with worsening disease severity (96–98), surpassing even the levels seen in bacterial ARDS (86, 99). Phenotypically, BAL neutrophils exhibit similar activation signatures-such as calprotectin expression and NET production-as their circulating counterparts (100). However, it remains unclear whether specific transcriptional clusters of circulating neutrophils are selectively recruited to the lungs or whether they infiltrate the infection site indiscriminately. ScRNA-seq of BAL samples identified 5 transcriptionally distinct neutrophil clusters in severe COVID-19 patients, particularly those expressing VEGFA, chemokine receptors, S100 proteins, and IFNs, which differentially impact COVID-19 severity depending on interactions with other immune cells (101). Mild COVID-19 is characterized by a robust response of debris-clearing monocytes and anti-viral T-cells, with IFN^active^ neutrophils being involved in viral clearance. Conversely, severe infection is characterized by a depletion of monocytes and T-cells that keep neutrophils in check, resulting in uncontrolled neutrophilic responses characterized by the overwhelming production of pro-inflammatory alarmins (e.g., S100A8/A9) and NETosis (101).

Histological examinations of lung autopsies from patients who succumbed to SARS-CoV-2 have corroborated these findings, revealing the dense presence of alarmin- and NET-producing neutrophils (102, 103). Notably, S100A8/A9 is key to aberrant neutrophil responses in COVID-19, driving exaggerated innate immune inflammation and uncontrolled pathological damage (104). Inhibition of S100A8/A9 has shown promise in attenuating COVID-19-related pneumonia in experimental models (104).

These findings provide valuable insights into the complexities of neutrophil responses in the pathogenesis of COVID-19. Further investigations are warranted to understand the functional impact of distinct neutrophil subsets and specific properties that have potential as therapeutic targets.

### 3.3 Neutrophil extracellular traps in acute COVID-19

The involvement of NETs in acute COVID-19 was hypothesized early in the pandemic (105–107) and subsequently confirmed by seminal studies conducted by Zuo et al. and Middleton et al. (79, 108). These investigations documented that plasma MPO-DNA complexes were found to be elevated in COVID-19 patients and correlated with disease severity, as evidenced by a higher sequential organ failure assessment (SOFA) score, incidence of intubation and death, and lower PaO_2_/FiO_2_ ratios (79, 108). Studies that followed expanded on the prognostic role of NETs, with extracellular DNA, cit-H3, and NE also being associated with clinical events and ICU admission (109, 110). Neutrophils isolated from the plasma of COVID-19 patients exhibit heightened basal NETosis, suggesting a predisposition towards NET formation (79). Furthermore, the serum of COVID-19 patients induced NETosis in control neutrophils (79), highlighting the systemic activation state induced by the virus.

A striking feature of severe COVID-19 lung disease that sets it apart from other viral pneumonia is the presence of distinctive vascular features, including widespread thrombosis and microvascular occlusion with secondary lobular ischemia (111). These vascular abnormalities are associated with endothelial activation and damage, which, in turn, trigger inflammation and thrombosis. Histopathological examination of post-mortem lung specimens from COVID-19 patients has revealed a dense neutrophilic infiltrate, neutrophil-platelet interactions, and the presence of NETs in pulmonary microthrombi (79, 102, 112, 113). These observations suggest that NETs contribute to the coagulopathy observed in severe COVID-19. Mechanistically, the web-like structure of NETs acts as a scaffold for the deposition of various clotting factors, promoting coagulation (114). Additionally, extracellular histones in NETs activate endothelial cells and platelets via TLR2 and TLR4 (115–119), and NET-associated proteases cleave and inactivate tissue factor pathway inhibitor, augmenting tissue factor activity (120). Skendros et al. demonstrated that neutrophils release NETs enriched in tissue factor, contributing to thrombosis in COVID-19 ARDS (121). Other than this study, the mechanisms by which certain NET components contribute to COVID-19 pathogenesis have been extrapolated from non-COVID studies; direct evidence elucidating the specific pathogenic roles of these components in COVID-19 is scare in contemporary literature.

Multiple stimuli of NETosis in COVID-19 have been identified. There is evidence that SARS-CoV-2 can directly infect neutrophils through ACE2 and TMPRSS2, triggering NET formation (121). Platelets are a major inducer of NETs, as exposing control neutrophils to platelet-rich plasma from COVID-19 patients is sufficient to stimulate NETosis (121). Platelets, which do not typically interact with neutrophils, become important inducers of NETs in the context of bacterial and viral infections (22, 122, 123), perhaps mediated by platelet factor-4 (PF4) (103), p-selectin or high-mobility group box 1 (HMBG1) (119). Autoantibodies against PF4, observed in severe COVID-19 and vaccine-induced thrombotic thrombocytopenia (VITT), are also capable of stimulating NET formation (124–126). Lastly, elevated levels of complement proteins, particularly C3 and C5, in COVID-19 provide another potential pathway for NETosis (121, 123, 127).

To gain a deeper understanding of NETs in COVID-19, future studies employing higher-resolution approaches are needed. Genetic or pharmacologic loss-of-function experiments targeting specific NET components could provide valuable insights into their precise pathogenic effects. It is important to consider the dichotomous nature of NETs, as they have been shown to play both beneficial and pathologic roles in various contexts. For instance, NETs mitigate the systemic spread of HIV-1 and Chikungunya virus infection (128, 129), with DNase-1 administration leading to disseminated infection (129). NETs can also promote healing through the degradation of pro-inflammatory molecules by their proteases (130). Importantly, degrading NETs as a therapeutic strategy can inadvertently worsen the cytotoxic/pro-inflammatory/pro-thrombotic effects of NET-bound DNA and histones when they are liberated from NETs as NET-degradation products (131, 132). NETs can suppress pro-inflammatory macrophage phenotypes in autoimmune disorders like rheumatoid arthritis (133), and induce anti-inflammatory M2 polarization in *Leishmania* parasite infection (134) and myocardial infarction (135), associated with decreased tissue damage in the latter. The balance between these dual roles may reflect factors such as the origin of neutrophil subsets, the stimuli triggering NET formation, the degree of neutrophil activation, and the composition of NETs themselves. Therefore, comprehensive investigations into NETs in COVID-19 are crucial for discerning their precise contributions and facilitating more precise therapeutic manipulations.

## 4 Neutrophils and NETs in chronic lung disease

Investigations into the association between neutrophil responses, NETs, and LC have predominantly focused on chronic lung disease and pulmonary fibrosis. The discussion herein discusses the role of neutrophils and NETs in chronic lung inflammation and pulmonary fibrosis in a myriad of non-COVID diseases.

### 4.1 NETs in chronic inflammation

NETs have emerged as pivotal contributes towards several chronic lung diseases, including asthma, chronic obstructive pulmonary disease (COPD), cystic fibrosis, and bronchiectasis, where they damage host tissue, impair mucociliary clearance, hinder bacterial cell killing, and exacerbate inflammation (136). Elevated neutrophil counts and NET markers, such as cell-free DNA, have been observed in the lungs and sputum of COPD and asthma patients, demonstrating a positive correlation with disease severity and the frequency of exacerbations (137–140). The release of the inflammasome-related cytokine IL-1β by macrophages can induce NETosis in asthma (141). NET contents components such as DNA, LL-37, defensins, and NE are pro-inflammatory and induce the release of histamine and leukotrienes, leading to worsening disease severity (136, 142).

Cystic fibrosis patients experience recurrent infections and have a lung microenvironment rich in pro-inflammatory cytokines and chemokines that promote neutrophil infiltration and NET production (143). The sputum of cystic fibrosis patients exhibits higher levels of extracellular DNA, NE, MPO, and calprotectin (144–146). Among these, DNA and NE are particularly associated with declining lung function and increased disease severity (147–149). Additionally, the virulence factor pyocyanin produced by *Pseudomonas aeruginosa—*the primary cause of pneumonia in cystic fibrosis patients—can enhance NET formation (150, 151). DNase is an effective therapy in reducing mucus viscosity in CF patients by degrading NET-derived DNA (52). However, the double-edged role of NETs is evident here, with NET degradation recently shown to liberate NET-bound NE, enhancing its activity and facilitating tissue damage (152).

Bronchiectasis is characterized by non-resolving neutrophilic inflammation and is marked by elevated levels of NETs in sputum, with their levels positively correlated with exacerbation incidence, worsening lung function, and mortality (153). Treating active infections or reducing inflammation in bronchiectasis patients reduces NET levels, leading to clinical improvement (154). Moreover, emerging evidence suggests that antibiotics such as macrolides can directly attenuate NETs independent of their antimicrobial effects (154).

Together, these findings underscore the role NETs play in lung diseases featuring chronic inflammation. Mechanistically, NET components contribute to the amplification of pro-inflammatory cytokines and chemokines, including IL-1, IL-6, and IL-8, by alveolar epithelial cells and alveolar macrophages, thereby perpetuating repetitive cycles of inflammation and alveolar epithelial damage (155, 156). Smoking-related airway disease exemplifies these features, as smoking induces the release of NETs, which, in turn, promote T-cell differentiation and pro-inflammatory cytokine secretion by airway epithelial cells and macrophages, ultimately fostering chronic airway disease (157, 158).

NETs also damage the alveolar epithelium in various models of acute lung injury, including transfusion-associated lung injury (159), trauma (160), ventilator-associated lung injury (161), primary graft dysfunction after lung transplantation (162), lipopolysaccharide-related sepsis (163), influenza pneumonitis (164), and COVID-19 (79, 108, 165, 166), all of which are known to feature fibrosis as a sequela. A recent review by Scozzi and colleagues details the role of NETs in these diseases (167). The induction of NETs leads to cytotoxic effects on the alveolar epithelium and vascular endothelium (146, 160, 168). Additionally, NETs contain tissue factor, which contributes to thrombosis in ARDS (169).

### 4.2 NETs in fibrosis

Interstitial lung disease (ILD) is an umbrella term that encompasses a range of conditions characterized by diffuse lung parenchymal changes, alveolar inflammation, and interstitial fibrosis. ILD can result from various causes, including environmental (pneumoconiosis, hypersensitivity pneumonitis), smoking, drugs (bleomycin, chemotherapy, nitrofurantoin), connective tissue disease (SLE, scleroderma), or idiopathic origins (IPF vs non-IPF). This discussion focuses on specific pathways through which NETs contribute to fibrosis, shedding light on potential mechanisms that may also translate in the context of LC.

Lung fibroblasts, which constitute the majority of the cellular constituents of the lung parenchyma, are the major effectors of pulmonary fibrosis (170). Several NET components have been associated with fibroblast activation (170). Myofibroblasts exposed to NETs display increased expression of connective tissue growth factors, collagen production, and proliferation/migration, with these effects being mitigated by DNase-1 treatment (171). Bleomycin, a drug known to induce pulmonary fibrosis, triggers PAD4-dependent NET formation in neutrophils, contributing to the development of ILD. Accordingly, PAD4-*KO* mice display attenuated bleomycin-induced NETosis with decreased pulmonary fibrosis (172).

NET components such as histone H3 and MPO promote the differentiation of lung fibroblasts into myofibroblasts, an effect that is attenuated by exogenous DNase-1 administration (173). NE within NETs has recently been shown to induce fibroblast proliferation and myofibroblast differentiation, thereby facilitating the progression of lung fibrosis (174). Notably, NE-*KO* mice were protected from asbestos-induced lung fibrosis (174). NE has also been implicated in promoting α-SMA and fibronectin expression in macrophages, resulting in a fibrotic phenotype termed the macrophage-to-myofibroblast transition (175, 176). Additionally, IL-17 contained within NETs enhances the fibrotic activity, but not the differentiation, of myofibroblasts, suggesting that the NET components mentioned earlier may prime the process of IL-17-driven fibrosis (171).

Epithelial-to-mesenchymal transition (EMT), induced by NETs, has been observed in LC and is a widely recognized phenomenon in various disease contexts. Chronic lung allograft dysfunction (CLAD), characterized by marked lung fibrosis and subsequent dysfunction, is associated with NET production, which triggers EMT in alveolar epithelial cells, as evidenced by increased α-SMA and decreased E-cadherin expression, closely resembling the effects of the well-known EMT inducer TGF-β (177). Endothelial cells can internalize NETs through their surface RAGE receptors (178), but the persistent NET release overwhelms their uptake capacity, leading to the accumulation of NETs in the extracellular space. Subsequently, NE disrupts intercellular tight junctions to increase vascular permeability and promotes endothelial-to-mesenchymal transition by promoting β-catenin signaling (178). Macrophages, also through RAGE receptors, can internalize NETs (176). The phagocytosis of NE into macrophages has been shown to induce the macrophage-to-mesenchymal transition in murine models of post-spinal surgery fibrosis (176). Similarly, NETs facilitate the macrophage-to-myofibroblast transition and attenuate TGF-β1 secretion from macrophages, promoting renal fibrosis after unilateral renal obstruction and the fibrotic remodeling of chronic venous thrombi (179, 180).

## 5 Neutrophil extracellular traps in long covid

### 5.1 The theorized role of NETs in long COVID

Sawadogo et al., drawing upon knowledge of NETs and their interaction with the adaptive immune system, hypothesized a link between prolonged neutrophil activation, NET release, and the development of LC (181). They highlighted the fact that NET components—including double-stranded DNA, histones, citrullinated peptides, MPO, and proteinase-3—are unbeknownst to the adaptive immune system and, hence, constitute neoantigens. These neoantigens have the potential to initiate and sustain autoimmune processes by triggering the production of autoantibodies (14, 182). Compelling evidence from conditions like systemic lupus erythematosus and rheumatoid arthritis shows that the presence of autoantibodies can induce NETosis and subsequently protect them from degradation, supporting the notion that NETs can foster autoimmunity by harboring neoantigens (182). This phenomenon would fuel a chronic pro-inflammatory response, activation of the coagulation cascade, and fibrosis (40, 82). Similar to SLE, COVID-19 exhibits the presence of LDNs in circulation, which release NETs enriched with oxidized nucleic acids that possess heightened immunostimulatory capabilities, further enhancing autoimmunity and IFN responses (27). Our group similarly postulated a role for NETs-related autoimmune vasculitides as a mechanism of LC vascular disease (183).

**Figure 2:**
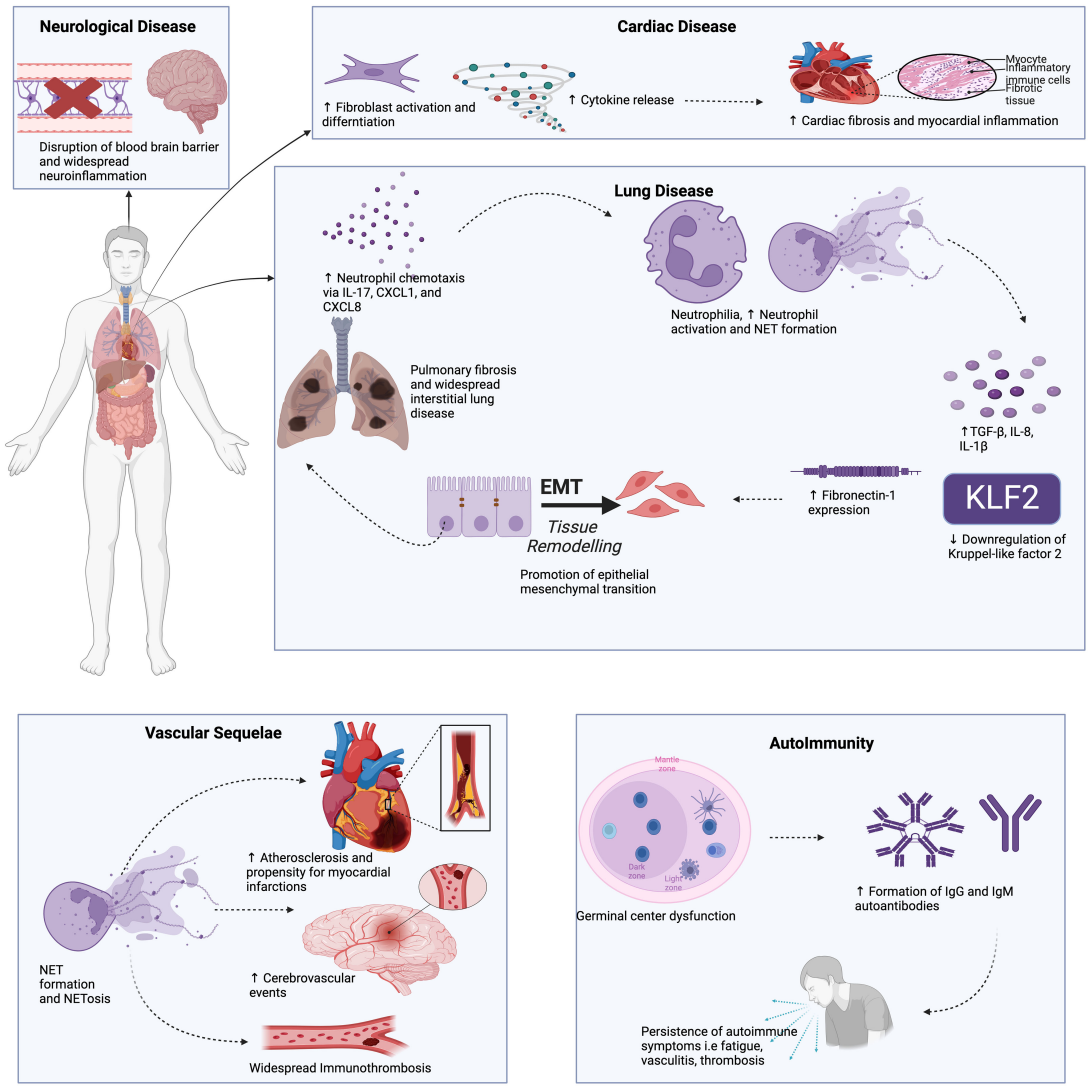
The potential role of NETs in long COVID may involves multiple pathways. NETs have been shown to drive pro-fibrotic responses in the alveolar epithelium and lung fibroblasts, leading to lung fibrosis. An array of autoantibodies detected in long COVID, including anti-NET IgM and IgG, which have been shown to protect NETs against degradation, contributing to downstream inflammation, vascular damage, and thrombosis. NETs are known to contribute to atherosclerosis and myocardial inflammation, perhaps explaining the cardiovascular sequelae in long COVID patients. Neuropsychiatric abnormalities in long COVID are associated with neuroinflammation and blood-brain barrier disruption, which can be induced by neutrophil-endothelial cell interactions and NET production. This Figure was created using BioRender.com.

The discussion below focuses on the role of NETs in autoimmunity, lung disease, cardiovascular disease, and neurologic/neuropsychiatric complications seen in LC (**Figure 2**). We also present emerging data showing cellular senescence-associated inflammation to play a role in LC pathogenesis.

### 5.2 NETs and autoantibodies in long COVID

Studies have demonstrated that circulating neutrophils isolated from individuals with LC display higher levels of NET induction compared to those of healthy controls (184, 185). Longitudinally following surrogate NET markers for at least 6 months in patients previously hospitalized for COVID-19 demonstrates sustained elevations in serum concentrations of NE, MPO, and cell-free DNA, although they were lower compared to acute COVID-19 patients but still higher than non-COVID controls (186). These findings indicate an incomplete resolution of NETs after acute COVID-19.

Severe COVID-19 features the emergence of autoantibodies against a wide array of proteins including type I IFNs, numerous interleukins, and self-antigens associated with autoimmune diseases, such as anti-nuclear antibodies (ANAs), anti-histone antibodies, and anti-neutrophil cytoplasmic antibody (ANCA), which are components of NETs (187–190). Most of the autoantigens identified exist complexed to extracellular nucleic acids—and such would be the case within NETs—which are then recognized by nucleic acid PRRs such as Toll-like receptors (e.g., TLR7) (189). Although the exact mechanism of their pathogenicity is unclear, their presence is positively correlated with disease severity. However, a large cohort study comparing non-ICU and ICU-admitted COVID-19 patients demonstrated that ANAs are not associated with disease severity but rather reflect a dysregulated immune response due to extensive cell death (191).

Importantly, the detection of IgG and IgM autoantibodies against NETs is frequent in hospitalized COVID-19 patients, their levels tracking with increased circulating NET markers and worse disease outcomes (192). The levels of anti-NET IgG inversely correlate with the ability to clear NETs, suggesting that autoantibodies protect NETs from degradation by DNase-1 (192). Similar observations have been made in SLE, where anti-dsDNA and anti-histone antibodies are thought to protect NETs against degradation by circulating DNase (193).

Mechanistically, after an infection, the germinal center B-cell response is crucial for the development of high-affinity antibodies through the processes of class-switching, somatic hypermutation, and affinity maturation. Impaired germinal center B-cell responses, as observed in severe and critical COVID-19 cases, lead to the emergence of extrafollicular B-cell responses (194, 195). This aberrant activation pathway induces the production of polyreactive autoantibodies with limited somatic hypermutation, similar to patterns observed in SLE (196). These polyreactive antibodies, along with an abundant supply of self-antigens from dead or dying cells and NETs, may drive the development of autoantibodies in COVID-19 (197).

In the context of LC, COVID-19 survivors exhibit higher detectable levels of ANAs 3 months and 12 months post-infection compared to age- and sex-matched healthy controls (198). Persistently positive ANA titers are associated with LC symptoms of fatigue, dyspnea, and cough (198). ANA positivity also correlates with higher levels of TNF-α (198). The presence of autoantibodies associated with antiphospholipid syndrome, including anti-cardiolipin, anti-phosphatidylserine/prothrombin, and anti- β_2_ glycoprotein, is evident in approximately half of hospitalized COVID-19 patients (199). Moreover, these IgG autoantibodies induce NETosis in neutrophils isolated from healthy patients and accelerate venous thrombosis when injected into mice (199). More recently, it was demonstrated that a significant fraction of LC patients exhibit positivity for IgM/IgG anti-cardiolipin and anti-β_2_ glycoprotein autoantibodies (186). In the same cohort, LC patients demonstrated higher levels of NET markers than healthy controls, indicating incomplete resolution of NETs after recovery from SARS-CoV-2 (186).

These findings draw intriguing parallels between COVID-19 and autoimmune diseases like SLE, highlighting the presence of NETs as a potential source of neoantigens. Dysregulated humoral responses, coupled with the sustained presence of NETs, contribute to the initiation and perpetuation of autoimmunity. However, discerning the pathogenicity of specific autoantibodies in LC remains largely unchartered territory, as existing data does not establish causal relationships.

### 5.3 NETs in long COVID lung disease

Respiratory symptoms such as cough and dyspnea are frequently observed in individuals with LC (200, 201). A meta-analysis of over 257,000 COVID-19 patients reported that dyspnea persisted for > 12 months after the initial COVID-19 infection in 31% of cases (202). Radiologically, a combination of persistent inflammation (ground glass opacities and consolidation) and fibrosis (fibrotic bands, interlobular septal thickening, and honeycombing) can be observed (203, 204). Persistent inflammation dominates the early post-acute phase, with fibrosis becoming more prevalent during the follow-up period (203). Significantly, approximately 45% of severe COVID-19 survivors will develop pulmonary fibrosis (205). Risk factors for the development of pulmonary fibrosis as LC include various indicators of more severe disease, such as ICU admission, mechanical ventilation, longer hospitalization, and steroid/immunoglobulin treatment (205).

One study by George et al. study has provided substantial insight into the role of NETs in the pathogenesis of LC lung disease, particularly by including a comparison group of patients who experienced complete clinical and radiologic resolution of acute COVID-19 symptoms (206). The authors demonstrated that a subset of severe COVID-19 survivors developed interstitial lung disease (ILD)-related changes on chest computed tomography scan at 3-6 months post-SARS-CoV-2 infection, accompanied by a restrictive pattern on pulmonary function testing. Patients with persistent interstitial changes exhibited significantly elevated neutrophil counts and serum MPO concentrations compared to controls, which positively correlated with the radiologic extent of pulmonary disease. Comparing the plasma proteome of these patients revealed that the neutrophil chemoattractant IL-17 was the only protein significantly associated with persistent ILD on multivariate analysis (OR 3.72, 95% CI 1.20-16.84, p=0.0403) (206). Furthermore, neutrophil chemokines CXCL1 and CXCL8 positively correlated with the degree of restrictive disease on pulmonary function testing, while CXCL8 and the inflammasome-related cytokine IL-18, along with its receptor IL-18R1, were directly associated with the radiological extent of interstitial disease (206). These differences were also reflected in nasal brushing samples taken to model mucosal immunological changes in the upper airways. *In vitro* experiments using alveolar epithelial cell lines showed that purified NETs increased the expression of fibronectin-1, vascular endothelial growth factor, and alpha-smooth muscle actin, while reducing E-cadherin expression, indicating that NETs drive epithelial-to-mesenchymal transition (EMT) and subsequent extracellular matrix deposition, which are established processes in pulmonary fibrosis (206). These changes were primarily driven by the host response to infection, such as through NETs, rather than direct viral infection of alveolar epithelial cells (206).

These findings are consistent with lung autopsies of deceased COVID-19 patients revealing the presence of alveolar epithelial cells co-expressing mesenchymal markers (207). *In vitro* experiments demonstrate that co-culturing alveolar epithelial cells with neutrophils, alveolar macrophages, and SARS-CoV-2 virus results in the production of factors such as TGF-β, IL-8, and IL-1β by alveolar macrophages, along with NET production, ultimately leading to a complete EMT signature. Notably, removing either neutrophils or alveolar macrophages resulted in an incomplete EMT phenotype (207). These findings support the notion of an alveolar macrophage/neutrophil/NETosis axis, whereby factors released by alveolar macrophage-derived factors induce NETosis, which, in turn, promotes EMT in pneumocytes.

Another study explored the role of Kruppel-like factor 2 (KLF2) in pulmonary sequelae of LC (208). KLF2 is a fibroblast protein, and its downregulation has been implicated in fibrosing disorders (209, 210). Lung fibroblasts stimulated with plasma from severe COVID-19 patients downregulate KLF2 and acquire a pre-fibrotic phenotype (208). Treating lung fibroblasts with a combination of DNAse-1 (to degrade NETs) and JAK/IL-6 inhibitors baritinib/tocilizumab (to attenuate inflammation) normalized KLF2 expression. Significantly, COVID-19 patients treated with this combination showed better outcomes compared to those receiving standard-of-care therapy. Furthermore, exposing lung fibroblasts to the plasma of treated patients resulted in higher KLF2 expression (208).

Together, these findings suggest that NETs and the inflammatory environment in the circulation and lung parenchyma of COVID-19 patients, particularly in severe cases, induce fibrotic phenotypes in alveolar epithelial cells and lung fibroblasts, which may explain the development of pulmonary fibrosis observed in a significant proportion of severe COVID-19 survivors.

### 5.4 NETs in the cardiovascular manifestations of long COVID

Several prospective and retrospective studies have consistently demonstrated a higher incidence of vascular pathologies, such as arterial thrombosis, venous thrombosis, atherosclerosis, vasculitis, and hypertension, in patients with LC (211–214). Notably, a prospective study of 153,760 COVID-19 patients revealed that convalescent individuals had a significantly higher risk of developing future cardiovascular disease, cerebrovascular disease, thromboembolic events, and ischemic heart disease compared to healthy contemporary and historical controls (211). This highlights a distinctive vascular feature of LC, characterized by widespread activation of pro-coagulant pathways (215–218). Indeed, elevated levels of pro-inflammatory and pro-thrombotic mediators have been observed in LC patients compared to healthy controls (219). It has recently been shown that NETosis persists at a greater level in LC patients compared to convalescent recovering patients (184). Persistent activation of pathways related to immunothrombosis and neutrophil activation has also been observed in COVID-19 survivors 6 months after the initial SARS-CoV-2 infection (220).

NETs are critical mediators of immunothrombosis and endotheliitis, exerting their function through various mechanisms (221, 222). NETs have also been implicated in the development of atherosclerosis, where endothelial cells promote NET formation. In turn, NETs lead to an increase in pro-inflammatory signaling and subsequent recruitment of immune cells to atherosclerotic plaques (223–228). NETs have also been shown to participate in the pathophysiology of ANCA-associated vasculitides (229), suggesting a potential link between autoimmunity, NETs, and vasculopathy. Promising results have been observed with NET-targeted therapies in the treatment of vascular pathologies, further supporting the involvement of NETs (230–233). Additionally, a larger burden of NETs has been observed in the coronary thrombi of COVID-19 convalescent patients with ST-elevation myocardial infarction when compared to historical controls (12, 234). These findings provide evidence of plausible evidence of NETs in the pathogenesis of vascular disorders in LC.

Cardiac involvement is an archetypal feature of LC and encompasses a wide range of presentations, including cardiac inflammation, cardiac fibrosis, dysrhythmias, ischemic heart disease, and cardiac impairment (235, 236). Cohort studies utilizing cardiac magnetic resonance imaging in patients with LC have demonstrated evidence of impaired ventricular function in addition to increased cardiac edema and inflammation (237–239). Additionally, patients with LC demonstrated a heightened incidence of myocardial injury particularly in the form of myocardial fibrosis (240). The interplay between NETs and cardiac disorders in several viral and bacterial infections was recently reviewed (241). Clinical evidence for the involvement of NETs in COVID-19-induced cardiac inflammation has recently been established through autopsy reports from 21 SARS-CoV-2 infected individuals, showing the presence of NETs in all patients and its association with myocarditis and cardiac injury (242). Additionally, targeting NETs in a mouse model of COVID-19 through DNase I therapy resulted in the attenuation of cardiac injury (243). Neutrophils recruited to the heart via the cytokine midkine (MK), which then induces NETosis, contribute to the pathogenesis of myocarditis and cardiac inflammation (244). Targeting MK attenuates neutrophil infiltration and NET formation, associated with a reduction in ventricular systolic dysfunction and myocardial fibrosis (244). *In vitro* studies have highlighted a potential role for NETs in the development of cardiac fibrosis through enhancing fibroblast migration and promoting cardiac myofibroblast differentiation (245). These findings suggest a potential role for NETs in non-ischemic cardiac injury in LC.

### 5.5 NETs in neurologic manifestations of long COVID

Another prominent feature of LC is the array of neurological complications the sizeable chronic burden of which has been indicated by numerous studies (246–249). On a gross scale, a UK BioBank study analyzed brain MRI scans pre- and post-infection and observed a greater clinical burden of cognitive decline and radiological changes of reduction in brain size and gray matter atrophy affecting the hippocampus and orbitofrontal cortex in the post-infection group (250).

Mechanistically, direct viral invasion of the brain does not appear to be prominent except in very severe acute COVID-19 cases (7). Instead, neuroinflammation, micro-clots, and the activation of CNS-resident glial cells are hypothesized to be crucial mediators of LC-associated neurological sequelae (251). Experiments in ACE2-transgenic mice have indicated that even a mild SARS-CoV-2 respiratory infection raises systemic cytokine levels, such as CCL11, which can be neurotoxic by inducing reactive states in microglia (252). Indicators of glial cell reactivity are elevated amongst LC patients with persistent depressive symptoms (253). A study of 76 LC patients experiencing “brain fog” (encompassing headache, fatigue, malaise, and altered level of consciousness) was the first to objectively demonstrate COVID-19-associated BBB disruption by utilizing neurological biomarkers and dynamic contrast-enhanced magnetic resonance imaging (254). Sustained elevations in S100β, IL-8, TGF-β, and GFAP were observed in brain fog LC patients, indicating persistent inflammation. Importantly, the adhesion of peripheral blood mononuclear cells to brain microvascular endothelial cells was enhanced in patients with brain fog, and exposing endothelial cells to the serum of these patients triggered endothelial cell activation (254).

A distinctive feature of LC is the higher propensity for developing ischemic strokes (251). Lee et al. documented the presence of microvascular injury indicated by scattered microthrombi, endothelial activation associated with the adhesion of autoantibodies and complement proteins, and BBB disruption indicated by the perivascular presence of fibrinogen in brain autopsies of individuals who died suddenly with or after COVID-19 (255, 256). Co-localizing with fibrinogen were microglia/macrophages, CD8^+^ T-cells, and reactive astrocytes, with areas of neuronal loss observed from microglia phagocytosis (255, 256). Fibrin clots are also generally elevated in the blood of LC patients (216). A recent study associated elevated serum fibrinogen and D-dimer levels relative to C-reactive protein during acute admission with neurocognitive deficits 6 and 12 months after acute COVID-19, supporting the notion of an acute inflammatory response being responsible for the long-lasting neurological effects of LC (257). Together, these findings suggest that BBB disruption during acute COVID-19 leads to the spillover of fibrinogen which, along with propagating a hypercoagulable state and micro-clots (258), foster neurotoxic resident glial cell phenotypes that damage neurons in a subtle but debilitating manner with long-lasting neurocognitive consequences (see (259) for a detailed review on the neurological effects of fibrinogen).

The brain is typically devoid of neutrophils and NETs due to the integrity of the BBB (260). However, in scenarios where the BBB is compromised, neutrophils infiltrate and NETs are often visualized in brain tissue where they contribute towards ongoing neuroinflammation (260, 261). Recent studies proposed that COVID-19 facilitates BBB disruption by increasing the expression of matrix metalloproteinase-9 (MMP-9) which leads to basement membrane degradation (262, 263). The levels of NETs in the brain have been directly related to the degree of neuroinflammation in model organisms of Alzheimer’s disease, meningitis, ischemic stroke, and traumatic brain injury (264). That being said, direct evidence of the involvement of NETs in cerebral micro-clots and neuroinflammation in acute and LC is lacking. The fact remains that many of the autoantibodies (82), complement proteins (121), fibrinogen (265), Von-Willebrand factor (266), and platelets (267) that are hypothesized to be key to the micro-clot formation in LC and driving its neurological manifestations are known to be intertwined with neutrophil biology and NET production. Hence, directing research efforts toward identifying and tackling NETs may improve our understanding of the neurological sequelae of LC.

### 5.6 Cellular senescence and NETosis

Cellular senescence refers to a state of cell cycle arrest accompanied by the release of inflammatory molecules collectively termed the senescence-associated secretory phenotype (SASP) (268). Cellular senescence is associated with a chronic low-grade inflammatory state and is causally implicated in aging and various chronic diseases, including pulmonary fibrosis, neurodegeneration, and cardiovascular disease (269–277). Viral infections, including SARS-CoV-2, trigger a cellular stress response, culminating in the induction of senescence, termed virus-induced senescence (VIS) (278).

The intracellular signaling pathways mediating SARS-CoV-2—induced VIS have been extensively reviewed elsewhere (278). Suffice it to say that multiple studies have shown elevated levels of senescence markers in the upper and lower respiratory tract of COVID-19 patients, indicative of SARS-CoV-2 infection-related VIS, which results in the elaboration of a pro-inflammatory SASP that recruits and induces pro-inflammatory M1 phenotypes in macrophages (279–282). Notably, some samples with a high burden of senescent cells did not show detectable viral infection, suggesting that senescence can persist even after clearance of SARS-CoV-2 (278, 280). Linking senescence induction to COVID-19 pathology, Lee et al. demonstrated that supernatant from SARS-CoV-2-induced VIS cells can induce endothelial cell senescence or apoptosis, promote M1 macrophage polarization, activate platelets, trigger NET production, and accelerate thrombosis (280). Treating with senolytic drugs, which eliminate senescent cells by causing their selective apoptosis, reduced the levels of SASP-reminiscent pro-inflammatory cytokines in hamster models of SARS-CoV-2 infection. However, the histopathological impact on inflammation and thrombosis did not significantly improve with senolytic treatment (280).

In the context of LC, it is important to note that senescent cells are typically cleared by the immune system because of their chemoattractive SASP (283). However, in certain circumstances, such as tumorigenesis, senescent cells can persist and contribute to long-term disease recurrence by evading immune surveillance (284–286). Although data on whether senescent cells persist and drive LC phenotypes are currently lacking, ongoing studies have demonstrated SARS-CoV-2 infection-induced VIS of human brain organoids in corticothalamic neurons and GABAergic ganglionic eminence neurons, which are responsible for modulating neuronal circuitry and processing of sensory information (287). SARS-CoV-2 was shown to induce the loss of dopaminergic neurons in the brainstem responsible for coordination and consciousness, potentially explaining abnormalities in these processes in LC (287). However, multiple studies have suggested that direct SARS-CoV-2 infection of the brain likely plays an insignificant role in acute and long neuro-COVID (251).

Additionally, the downstream consequences of senescence induction and pro-inflammatory SASP elaboration—in terms of changes in neuronal function, synaptic plasticity, astrocyte or microglial activation, or the recruitment of circulating innate and adaptive immune cells—are currently unclear. The BBB disruption in LC does support the role of neutrophils and NETs, as is indicated by their role in other CNS pathologies that involve BBB disruption (261). Other than the data above, no studies directly observing senescent cell persistence and their roles in LC in other organ systems such as the lungs have not yet been conducted, but several hypotheses have been put forward (288, 289). Exploring the link between senescence and NETosis is particularly important given the rapid evolution of senolytic therapies into clinical trials and the feasibility of targeting senescent cells; such research may uncover therapeutic targets with great potential for translation into clinical applications.

## 6 Conclusions and outlook

The findings discussed provided valuable insights into the involvement of neutrophils and NETs in LC. However, there are still unanswered questions that, once addressed, could position NETs as viable targets for therapeutic interventions. Understanding the heterogeneity of neutrophils in LC, exploring the factors that sustain increased NETosis induction levels after SARS-CoV-2 resolution, and examining the impact of NETs on other organ systems, particularly the cardiovascular and nervous systems, are crucial areas to investigate. It is somewhat counterintuitive that short-lived neutrophils can exhibit a sustained activation response in LC, perhaps being influenced by alterations in effector phenotypes of long-lived cells or microenvironmental changes in the bone marrow (290). The impact of location must also be explored, i.e., considering compartmentalized/organ-specific pathophysiological differences, influenced perhaps by the potential role of persistent senescent cells and their influence on immune cell recruitment, is vital. Identifying factors—such as autoantibodies—that determine NET degradation capacity in the post-acute phase, as well as elucidating the specific NET components responsible for organ-specific pathology such as lung fibrosis, will enhance our understanding of LC. Addressing these uncertainties may pave the way for clinically applicable strategies aimed at targeting NETs, potentially alleviating this emerging health crisis.

## Author contributions

AS: Conceptualization, Methodology, Supervision, Writing – original draft, Writing – review & editing. MO: Writing – original draft, Writing – review & editing. IA: Writing – original draft. GA: Writing – original draft. HA: Writing – original draft. SA: Writing – original draft. BS: Writing – original draft. KA: Writing – review & editing. AY: Conceptualization, Writing – review & editing.
